# Effect of surface wettability on the interfacial adhesion of a thermosetting elastomer on glass†

**DOI:** 10.1039/d1ra05916e

**Published:** 2021-09-21

**Authors:** Ye Wang, Christopher J. Hansen, Chi-Chin Wu, E. Jason Robinette, Amy M. Peterson

**Affiliations:** Department of Plastics Engineering, University of Massachusetts Lowell Lowell MA 01854 USA amy_peterson@uml.edu; Department of Mechanical Engineering, University of Massachusetts Lowell Lowell MA 01854 USA; Weapons and Materials Research Directorate, US Army Combat Capabilities Development Command-Army Research Laboratory Aberdeen Proving Ground MD 21005 USA

## Abstract

Interfacial adhesion dictates properties and performance of both composites and adhesively bonded structures. Weak adhesion at the interfaces of polymer composites leads to void formation and debonding, which adversely affect composite structural integrity and mechanical performance. This work investigated the relationship between surface wettability and interfacial fracture energy with the goal of tailoring interfacial adhesion within polymer composites. A series of model functionalized surfaces was created using silane coupling agents with different organo-functionalities to alter surface wettability. Based on the analysis of interfacial fracture energy between a thermosetting elastomeric polymer network and model surfaces, interfacial adhesion was found to be positively correlated to resin wettability. The results provide a fast and simple approach to screen different material combinations for the development of novel polymeric composites and adhesively bonded structures with tailorable adhesion.

## Abstract 2

Interfacial adhesion is postively correlated with resin wettability.

## Graphical abstract

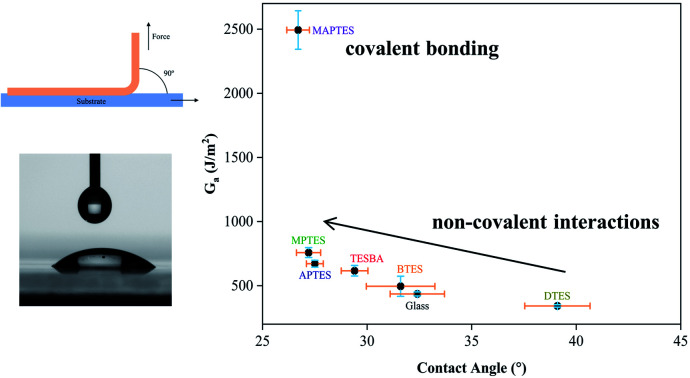

## Introduction

Interfaces within composites and adhesively bonded structures are essential to their function. For composites, interfacial interactions during processing can play an important role in the final composite structure, while interfacial adhesion during the service life of the composite is essential to load transfer from the polymer to the reinforcement phase. Similarly, interfacial interactions during fabrication of adhesively bonded structures affects microstructure and performance, and interfacial adhesion during use enables load transfer between adherends.

Silane coupling agents are commonly used to compatibilize reinforcements (*i.e.*, reinforcing phases) with continuous matrices through modifying the surface chemistry of the reinforcement.^1–4^ Silane agents at the interphase can act as bridging or bonding agents to modify the interfacial adhesion between the fibers and polymer matrices.^4–6^ Additionally, amine-containing silane coupling agents have been used to improve the tensile and flexural properties of polymer composites containing nanoparticles such as titania,^7^ silver,^8^ and nanodiamond.^9^

While tailoring of composite materials *via* reinforcement surface modification has been extensively studied, relatively fewer research efforts have been reported that focus on characterization of surface wettability in combination with interfacial adhesion of bulk composites. In one, Schultz and Lavielle studied surface properties of carbon fiber-epoxy matrix composites *via* inverse gas chromatography and found that fiber-matrix adhesion is positively correlated with increased acid–base interactions from interfaces.^10^ In another, Baillie *et al.* reported that increased surface acidity would positively affect the interfacial shear strength of carbon fiber-epoxy composites.^11^

In most of the previously discussed literature, the polymer phase has a glass transition temperature (*T*_g_) that is substantially higher than the room temperature, with a system that is designed to be in a glassy state for its entire service life. However, adhesion between rubbery polymers and stiff substrates is also an important area of investigation.^12,13^ Polymers with sub-ambient *T*_g_s are commonly used as adhesives, such as pressure sensitive adhesives (PSAs),^13–17^ hot melt adhesives (HMAs),^18–20^ and thermosetting adhesives.^21,22^ Kowalski *et al.* studied the tack properties for synthesized acrylic PSAs on polymer, stainless steel and glass substrates, and found that larger differences in surface energy between the PSAs and substrates led to increased tack.^17^ Similarly, Sowa *et al.* found that the peel adhesion strength of acrylic PSAs increased as the differences in surface energies between PSAs and substrates increased.^23^ Despite the large body of work in adhesion between soft materials and stiff substrates, we lack fast, simple methods for efficiently screening different combinations of materials with strong *versus* weak adhesion.

This work presents an investigation of the relationship between surface wettability and interfacial adhesion between an elastomeric polymer and stiff substrates. Several model functionalized glass substrates were prepared using silane coupling agents with different organo-functionalities and a thermosetting elastomeric acrylate was used as the polymer matrix. The surface functionality of silane-modified glass surfaces was confirmed after each step *via* advanced spectroscopy and the surface energy was determined using the Owens–Wendt–Rabel–Kaelble (OWRK) model. Interfacial adhesion between the model functionalized surfaces and the polymer network was characterized by 90° peel tests.

## Materials and methods

### Materials

Borosilicate glass surfaces, in either slide form (Fisher Scientific) or large plate form (McMaster-Carr), were used as the substrates for surface functionalization. Silane coupling agents with different organofunctional groups (Gelest) were all used as received for fabrication of model functionalized surfaces (Table 1). Ethanol (EtOH, Fisher Scientific) was used as received for hydrolysis and condensation of silane coupling agents onto the substrates. Ebecryl 230 (Allnex), isobornyl acrylate (IBA, Sigma-Aldrich), diphenyl (2,4,6-trimethylbenzoyl) phophine oxide (TPO, Sigma-Aldrich), and 2,5-bis(5-*tert*-butyl-benzoxazol-2-yl) thiophene (BBOT, Sigma-Aldrich) were used as received for the urethane acrylate resin system. Molded polytetrafluoroethylene (PTFE) bars were acquired from McMaster-Carr. All other chemicals in this work were used as received from Sigma-Aldrich.

**Table 1:** Silane coupling agents used to create model functionalized surfaces

Product name	Abbreviation	Chemical structures
Triethoxysilylbutyraldehyde	TESBA	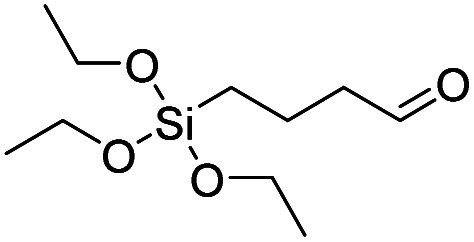
3-Mercaptopropyltriethoxysilane	MPTES	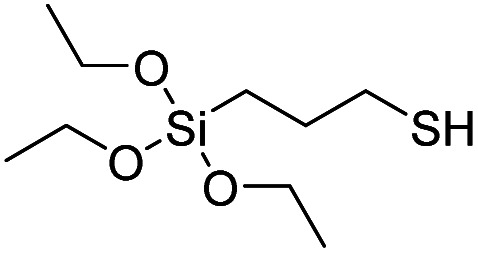
Methacryloxypropyl-triethoxysilane	MAPTES	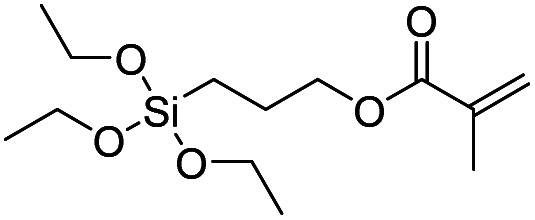
Benzyltriethoxysilane	BTES	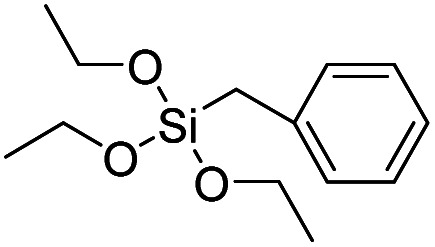
Docosyltriethoxysilane	DTES	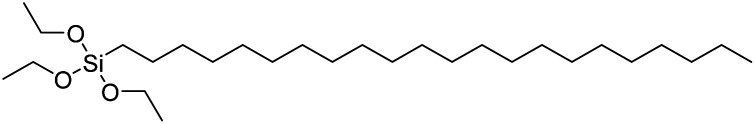
3-Aminopropyltriethoxysilane	APTES	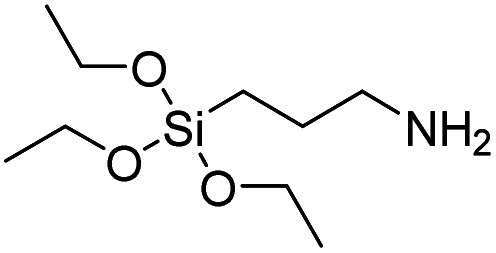

### Surface functionalization

Prior to functionalization, borosilicate glass surfaces (slides/plates) were cleaned by passing through a propane flame, followed by rinsing with acetone, ethanol, and DI water to remove any possible impurities. A 20 min cycle of UV–ozone cleaning was then performed in a UV ozone chamber (UV Ozone Cleaner-ProCleaner™ Plus, BioForce Nanosciences). Surface functionalization was achieved through hydrolytic deposition of silane coupling agents. Solutions consisting of 2 wt% of a silane coupling agent in a 95 : 5 by weight ratio of ethanol to water were prepared. For 3-mercaptopropyltriethoxysilane (MPTES), methacryloxypropyltriethoxysilane (MAPTES), and benzyltriethoxysilane (BTES) solutions, acetic acid was further added to adjust the solution pH to 4.5, based on the procedures provided by the supplier.^24^ All solutions were hydrolyzed for one hour at room temperature. Cleaned glass surfaces were immersed in hydrolyzed solutions for one hour, followed by rinsing with pure ethanol for 5–10 seconds, and placement in an oven at 110 °C for 30 minutes to achieve surface silanization. The solutions were continuously stirred using a magnetic stir bar at 100 rpm during hydrolysis and silanol formation.

### Surface characterization

Cleaned and functionalized surfaces were characterized using three methods: attenuated total reflectance-Fourier transform infrared (ATR-FTIR) spectroscopy, X-ray photoelectron spectroscopy (XPS), and contact angle measurements (sessile drop method). A Thermo Scientific Nicolet iS50 FT-IR with a diamond crystal was used for ATR-FTIR evaluation of glass and model functionalized surfaces. A Thermo Scientific K-Alpha+ XPS system with monochromatic soft aluminum *K*_α_ X-ray (1486.6 eV) was used to obtain the spectra and estimate the compositions of glass and functionalized surfaces. The samples were analyzed at 90° take-off angle under high vacuum (∼8 × 10^−8^ mBar). Core level lines for carbon, oxygen, silicon, nitrogen, and sulfur were calibrated with respect to the adventitious C1s at ∼284.8 eV (characteristic of C–C chemical state). Both survey scans and high-resolution spectra were collected and analyzed using Thermo Avantage control software.

A Biolin Scientific Theta Flex optical tensiometer was used for measuring static contact angles. Water, benzyl alcohol, glycerol, and chloroform were adopted as probe liquids, as they represent a wide range of polar and dispersive surface tensions, which can lead to more accurate calculations for the substrate surface energies. The Young's equation and the Owens–Wendt–Rabel–Kaelble (OWRK) method were applied, as described below, to determine glass substrate surface energies.^25–27^ For urethane-acrylate resins, surface tensions were determined *via* the pendant drop method.^28,29^ At the end of a pipette tip attached to the dispenser, a droplet of thermosetting resin was formed and was imaged *via* OneAttension (Biolin Scientific). The contour of each droplet and the radius of curvature were extracted to calculate the total surface tension of the resin. Each droplet of thermosetting resin was dispensed to be as large as possible (∼12.5 μL) because larger droplets can provide more reproducible, accurate, and precise results.^28^ The sessile drop method was then used in combination with the pendant drop method to determine the polar and dispersive components of resin surface energy. The PTFE molded bars, which have no polar components of surface energy, were used as the substrate for measuring static contact angles for the resin.

Surface energy and contact angles are related *via* Young's equation:

1 *γ*_sl_ = *γ*_sv_ + *γ*_lv_ cos *θ*

where *θ* is the contact angle and *γ*_sv_, *γ*_sl_, and *γ*_lv_ are the surface energies of the solid–vapor, solid–liquid, and liquid–vapor interfaces, respectively. Eqn (1) is used to describe the force balance at the 3-phase contact point of solid, vapor, and liquid phases. The OWRK theory is a commonly used method for determining surface energy due to its overall simplicity and good agreement with experimental results.^30–32^ The solid–liquid interfacial tension *γ*_sl_ is described as

2 *γ*_sl_ = *γ*_sv_ + *γ*_lv_ − 2(*γ*^d^_s_*γ*^d^_l_)^1/2^ − 2(*γ*^p^_s_*γ*^p^_l_)^1/2^

The d and p superscripts represent the dispersive and polar components of the total surface energy. Combining eqn (1) and (2), the OWRK theory can be further written as

3 *γ*_lv_ (1 + cos *θ*) = 2(*γ*^d^_s_*γ*^d^_l_)^1/2^ + 2(*γ*^p^_s_*γ*^p^_l_)^1/2^

Additionally, the polar and dispersive components are treated as additive components of surface energy in the OWRK theory, such that

4 *γ*_s_*= γ*^d^_s_*+ γ*^p^_s_

5 *γ*_l_ = *γ*^d^_l_ + *γ*^p^_l_

To calculate surface energy of different materials, static contact angle measurements are conducted with at least two liquids with known *γ*^d^_l_ and *γ*^p^_l_.

If the dispersive and polar components of surface energy (*γ*^d^_s_, *γ*^p^_s_) are given or determined for a solid, then the wetting behavior for different liquids can be simply predicted *via* the creation of a wettability envelope, which is the area enclosed within a contour of the polar and dispersive components of surface energy plotted in a Cartesian coordinate system. Any probing liquids with surface energies that fall within the enclosed area will wet the corresponding solid. Generally, polar and dispersive components of the probing liquid for which the contact angle is zero (cos *θ* = 1) are used for constructing wettability envelopes.^31,33,34^

### Resin curing and characterization

An aliphatic urethane acrylate resin system was used as the thermosetting polymeric network. This resin was a mixture of 60.00 wt% Ebecryl 230 and 39.44 wt% IBA. 0.40 wt% TPO was added as a photoinitiator and 0.16 wt% BBOT was added as a UV blocker. Before curing, the components of the thermosetting resin were mixed and degassed multiple times for 3 min each at 2000 rpm using a planetary centrifugal mixer (Thinky, ThinkyMixer ARE-310). The resin was UV cured *via* direct exposure to a 400 W bench UV flood lamp (EC-2000, DYMAX) for 6 min.

Differential scanning calorimetry (DSC) was performed with a Discovery DSC (TA Instruments). Each individual measurement was carried out under a constant nitrogen flow on a small (3–7 mg) cured sample sealed in a Tzero DSC pan. A sealed sample pan was then placed in the DSC cell and cooled to −90 °C. The cell was then heated to 80 °C at a rate of 10 °C min^−1^.

Dynamic mechanical analysis (DMA) was performed using a DMA Q800 (TA Instruments) in single cantilever mode. Constant heating rate experiments were carried out at a heating rate of 3 °C min^−1^ from −100 °C to 100 °C, with an amplitude of 15 μm and a frequency of 1 Hz.

### Sample curing and mechanical testing

Samples for 90° peel tests were fabricated in accordance with ASTM D6862, by curing the urethane acrylate resin onto the substrates (both cleaned and model functionalized surfaces). The resin composition as well as the mixing and degassing protocol are described in the resin curing and characterization section. The mixed resins were then poured into a CNC-machined aluminum mold with precut silicone rubber sheets (Fig. S1†) and exposed to the 400 W bench UV flood lamp for 6 min of UV curing. The segmented mold was designed for fabrication of specimens with dimensions of 203.2 mm (*L*) × 25.4 mm (*W*) × 3.175 mm (*T*). Cured specimens were gently removed from the mold and rinsed with isopropyl alcohol before returning the specimens to the UV system for post-curing under UV exposure for an additional 6 min. The cured elastomer failed in the grips in initial investigation for the MAPTES-functionalized samples, so a layer of MAPTES-functionalized glass fiber was added to the mold during peel sample fabrication. More details on this modification are provided in the ESI.†

The 90° peel tests were performed on a dual column mechanical testing machine (Instron 5966) with a 10 kN static load cell and peel test fixture at a constant extension rate of 2.54 mm s^−1^. The strain energy release rate can be expressed as

6 *G* = *F*/*b*(1 − cos θ)

where *θ* is peel angle, *F* is peel force, and *b* is the width of any sample.^35^ For θ = 90, eqn (6) can be further simplified as *G* = *F*/*b*. Under steady-state crack propagation, the interfacial fracture energy *G*_a_ (also called interfacial fracture energy) is equal to the strain energy release rate *G*. Interfacial fracture energies were calculated from the mean values of plateau peeling forces divided by the sample width.

## Results and discussion

### Surface functionality

**Fig. 1:**
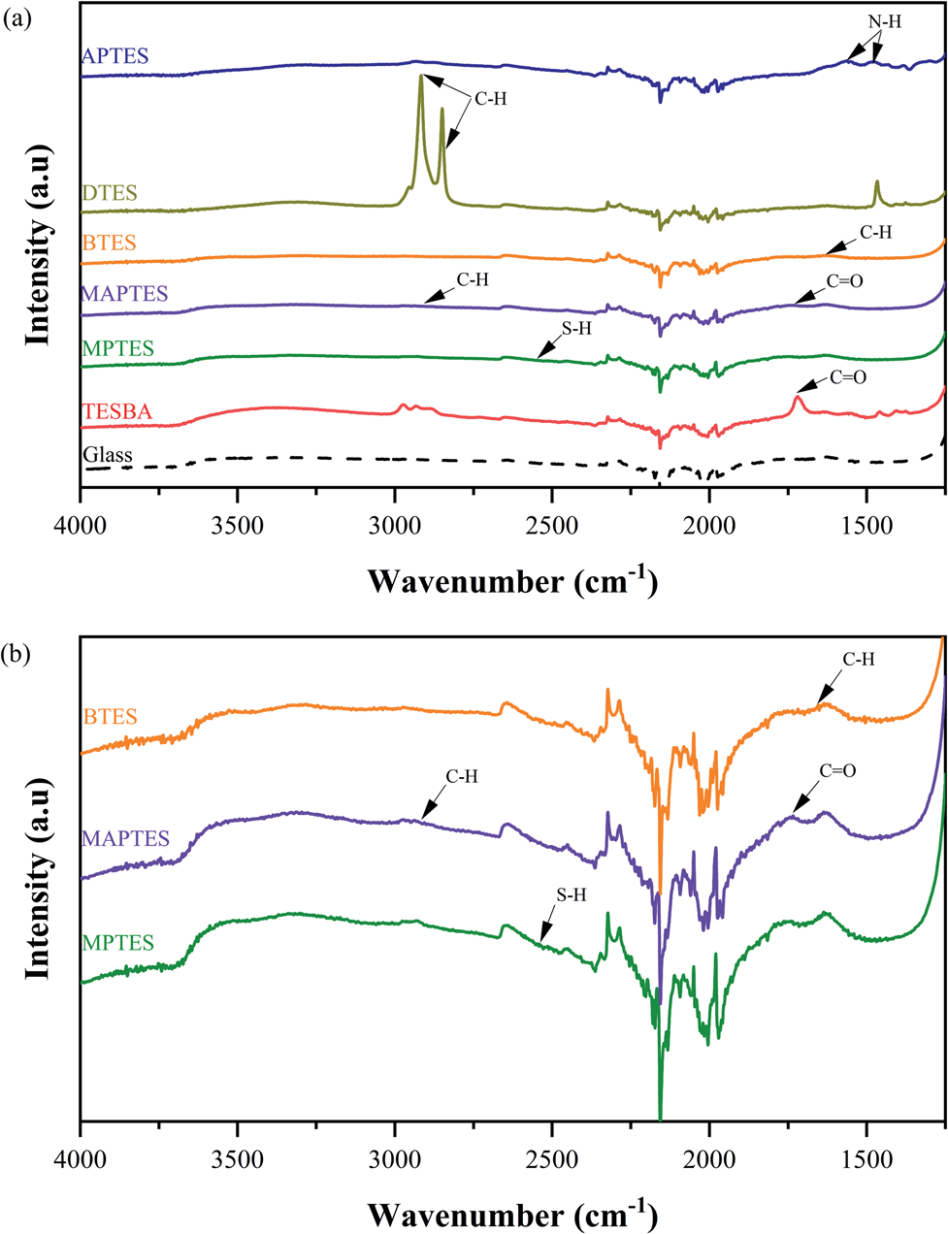
ATR-FTIR spectra of (a) plain glass and model functionalized surfaces, and (b) ATR-FTIR spectra with enlarged scale for MPTES-, MAPTES-, and BTES-functionalized surfaces.

Glass and model functionalized surfaces were first characterized *via* ATR-FTIR. Spectra for plain glass and surfaces functionalized with triethoxysilylbutyraldehyde (TESBA), MPTES, MAPTES, BTES, docosyltriethoxysilane (DTES), and 3-aminopropyltriethoxysilane (APTES) are presented in Fig. 1. In general, the various surface functionalizations for TESBA, MAPTES, DTES, and APTES are confirmed. However, for MPTES- and BTES-functionalized surfaces, the characteristic peaks are difficult to observe in ATR-FTIR spectra.

The APTES-functionalized surfaces exhibit peaks between 1400 and 1600 cm^−1^, which are consistent with N–H peaks from the amine groups, confirming surface functionalization.^36,37^ The strong peaks between 2800 and 3000 cm^−1^ in DTES-functionalized surfaces are characteristic of C–H stretching from the long alkyl chains.^38^ The absorption peak (Fig. 1b) for MAPTES at approximately 2900 cm^−1^ is attributed to C–H stretching, and the band around 1720 cm^−1^ is associated with C=O stretching.^39^ The TESBA-functionalized surfaces exhibit a strong peak between 1715 and 1740 cm^−1^ that is characteristic of C=O stretching and has previously been observed for TESBA-functionalized indium tin oxide nanoparticles.^40^

However, the spectra for BTES- and MPTES-functionalized surfaces are more difficult to interpret. The weak peak (Fig. 1b) between 2500 and 2600 cm^−1^ likely represents S–H stretching from the thiol groups of MPTES-functionalized surfaces.^41^ For BTES-functionalized surfaces, the peak at approximately 1650 cm^−1^ is consistent with C–H bending from the aromatic ring. These inconclusive results could be due to incomplete surface coverage. ATR-FTIR results qualitatively confirm the availability of the characteristic groups from TESBA, MAPTES-, DTES-, and APTES-functionalized surfaces but not the MPTES- and BTES-functionalized surfaces.

**Fig. 2:**
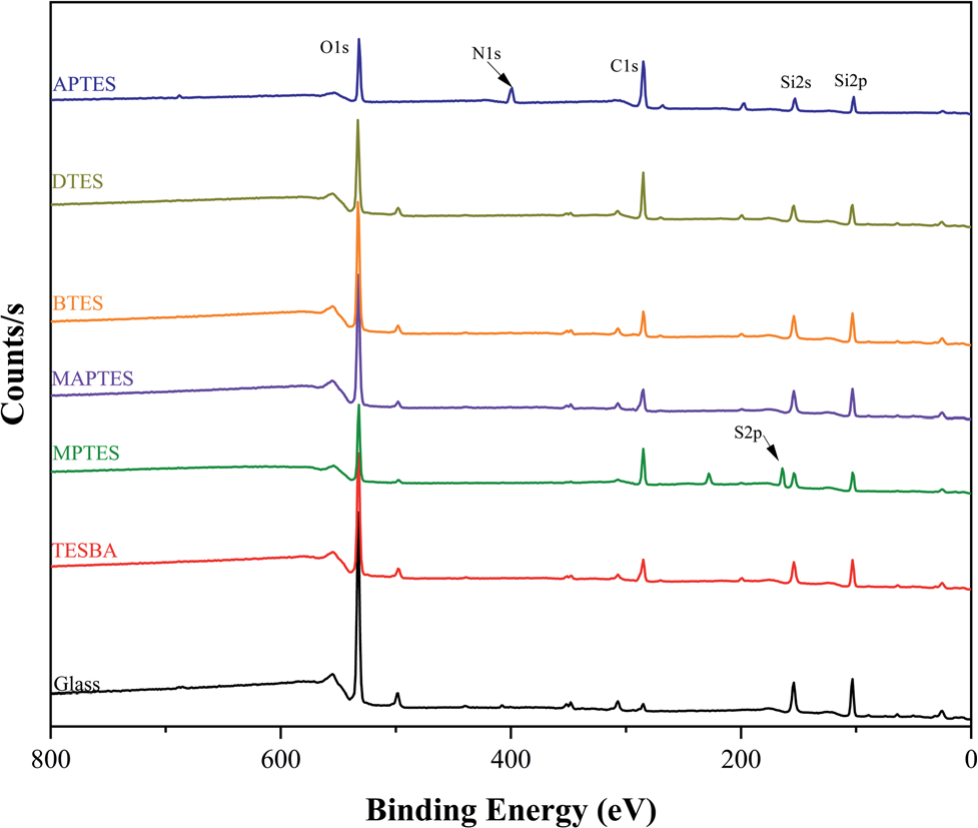
XPS survey spectra of glass and model functionalized surfaces.

Representative XPS spectra from surveys scans for plain glass and model functionalized surfaces are presented in Fig. 2. The survey scans show the presence of carbon, oxygen, and silicon for all the surfaces, as well as sulfur for MPTES-functionalized surfaces and nitrogen for APTES-functionalized surfaces with corresponding atomic compositions listed in Table 2.

**Table 2:** Summary of XPS atomic analysis results. Values presented in the table are atomic percent of the total composition

	C	O	Si	N	S	Other	C/O
Glass	6.94	60.85	26.22	—	—	5.99	0.111
TESBA	23.79	48.8	22.96	—	—	4.45	0.491
MPTES	44.08	25.91	19.66	—	8.04	2.31	1.701
MAPTES	23.30	51.64	22.80	—	—	2.26	0.451
BTES	20.01	50.01	24.83	—	—	5.16	0.401
DTES	38.88	37.25	19.44	—	—	4.43	1.041
APTES	48.57	22.64	13.19	11.89	—	3.71	2.151

A perfect APTES monolayer would have a C/N ratio of 3 : 1, consistent with full hydrolysis and condensation. In this work, the APTES-functionalized surfaces exhibit a C/N ratio of 4.1 : 1. This slightly higher C/N ratio is commonly observed for APTES-functionalized solids from previous studies and indicates partial silane coupling agent hydrolysis and condensation, which increases the relative amount of carbon.^42,43^

**Fig. 3:**
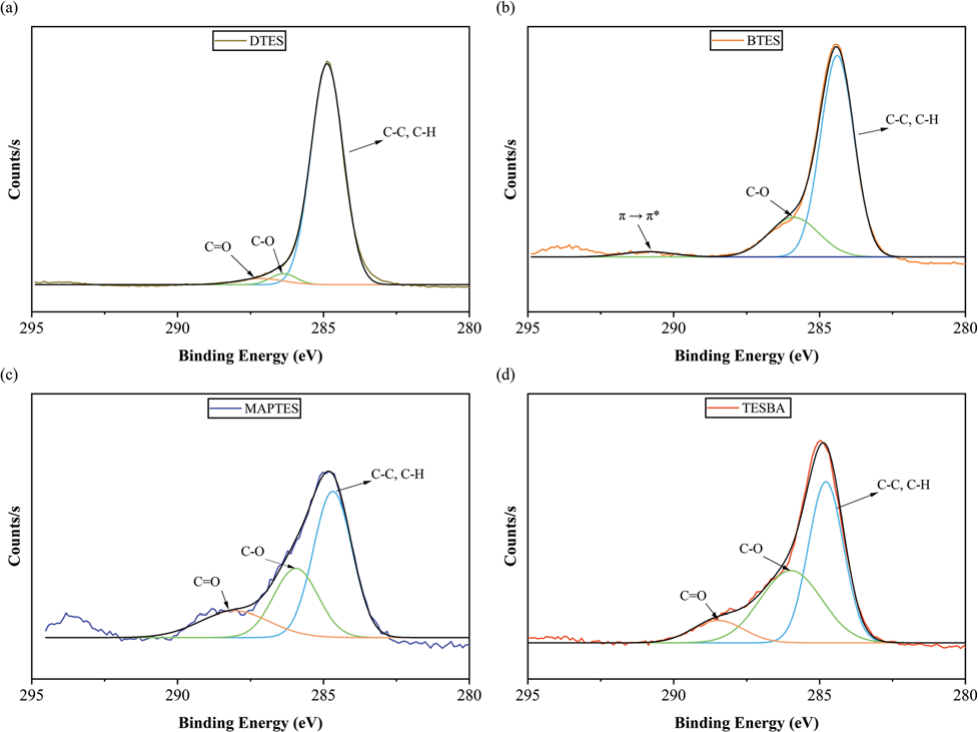
C1s XPS high resolution spectra of model functionalized surfaces with (a) DTES, (b) BTES, (c) MAPTES, and (d) TESBA.

A high fraction of carbon is expected for DTES-functionalized surfaces. Incomplete surface coverage was strongly indicated by its low C/O ratio of only 1.04 : 1 and high the Si content (19.44%). Further investigation of the high resolution C1s peak (Fig. 3a) shows that 90.82% of the measured carbon can be attributed to C–C and C–H, which is consistent with functionalization of silanes with long alkyl chains. These results are comparable to previous results from Ouyang *et al.*, who reported a C–C and C–H concentration of 90.26% for a dodecyltrimethoxysilane (DTMS)-functionalized wood fiber surface.^38^

For BTES-functionalized surfaces, the aromatic ring leads to characteristic π–π* satellite structures, as reflected near 291.2 eV in Fig. 3b,^44,45^ along with the C–C and C–H peak at approximately 284.6 eV, and a C–O peak around 286.1 eV. Similar to the DTES-functionalized surface, the higher content of oxygen and silicon measured suggests incomplete surface coverage.

The carbon, oxygen, and silicon compositions for the MAPTES-functionalized surface are significantly different from those of plain glass. The C/O ratio is 0.45 : 1, consistent with Dong *et al.* (C/O = 0.48 : 1).^46^ The deconvoluted C1s spectrum for MAPTES-functionalized surfaces shows C–H/C–C, C–O, and C=O bonds, as shown in Fig. 3c. Mitchell *et al.* evaluated the deposition of γ-methacryloxypropyltrimethoxysilane (MAPTS) on SiO_2_ surfaces and reported a broad C1s peak with C=O (18%), C–O (28%), and C–C/C–H (54%),^47^ consistent with our data.

As shown in Fig. 2, a peak corresponding to S2p is observed for the MPTES-functionalized surfaces at approximately 164 eV. An ideal MPTES monolayer, in which all the ethoxy groups are fully converted to siloxanes, should have a C/S ratio of 3 : 1. However, the C/S ratio is 5.5 : 1, which is within the range of previous published results (4.0 : 1 to 6.4 : 1) and suggests incomplete hydrolysis and condensation of the MPTES.^48–50^

For TESBA-functionalized surfaces, carbon and oxygen are detected *via* XPS, consistent with the ATR-FTIR results where characteristic C=O peaks are observed after surface functionalization. The deconvoluted high resolution C1s spectrum of TESBA-functionalized surface is similar to aldehyde-modified surfaces in previous studies.^51,52^ Based on the combined results from ATR-FTIR and XPS, surface chemistry of all the model functionalized surfaces has been confirmed.

### Surface energy and wettability

**Fig. 4:**
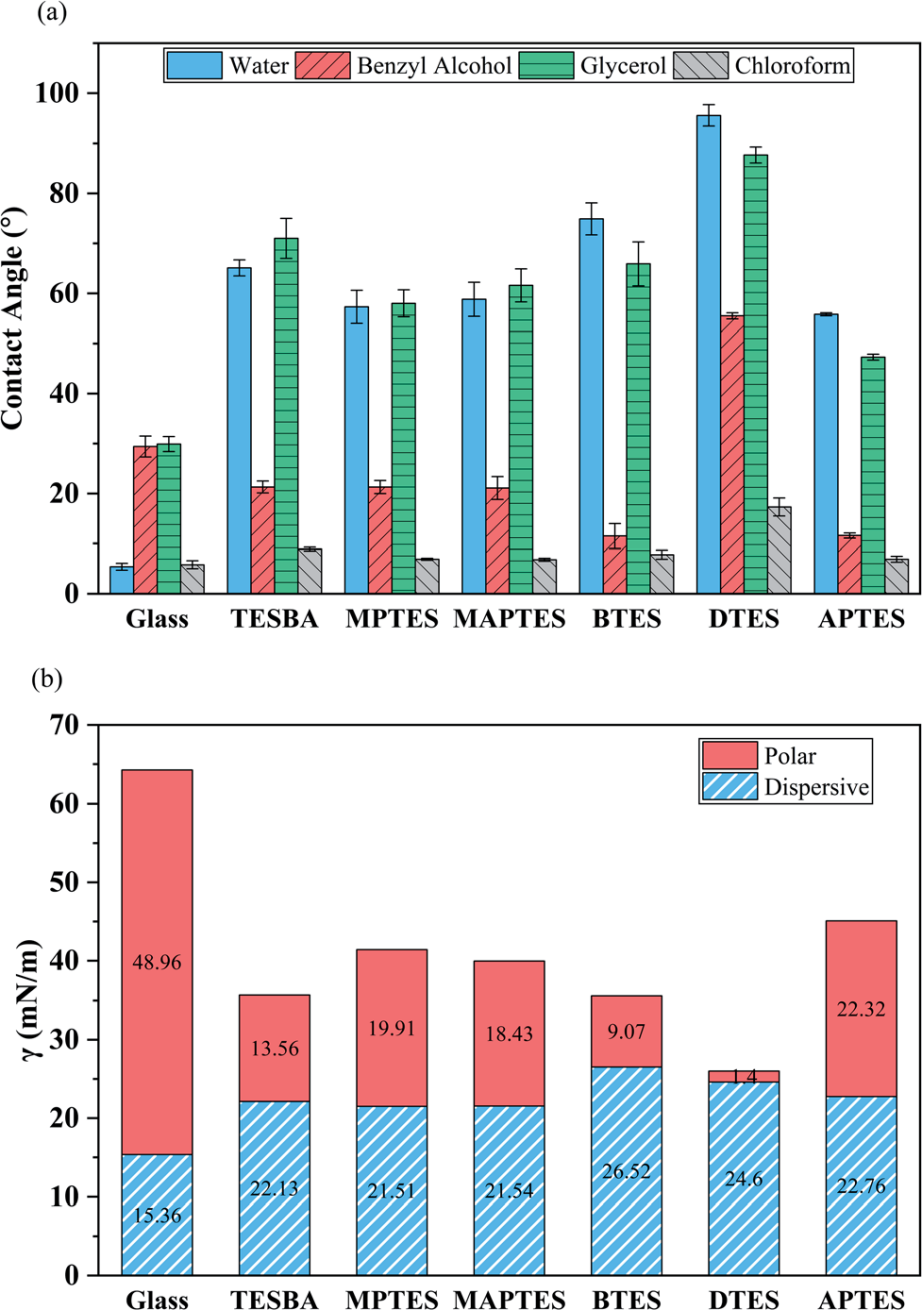
(a) Static contact angles for probe liquids on glass and model functionalized surfaces. Error bars represent standard deviations. *n* = 10 for each condition. (b) Surface energies, including polar and dispersive components, of glass and model functionalized surfaces.

Contact angle values for glass and model functionalized surfaces are shown in Fig. 4a. As anticipated, the surface wettability is dependent on surface functionalization. The water contact angles of glass and surfaces functionalized with TESBA, MPTES, MAPTES, BTES, DTES, and APTES are 5.4°, 65.1°, 57.3°, 58.8°, 74.9°, 95.6°, and 55.8°, respectively, which are consistent with literature values.^31,47,49,53–56^ Also included in Fig. 4a are the measured contact angles for other probe liquids. Chloroform contact angles on glass and model functionalized surfaces are the lowest since chloroform has the lowest surface tension of the four probe liquids. Contact angles of all probe liquids on DTES-functionalized surfaces remain the highest due to the presence of long alkyl chains. For other functionalized surfaces, variations of contact angles are due to the existence of organofunctional groups with different polarities. These data are used for determining surface energies and wettability envelopes.

Surface energies, shown in Fig. 4b, are calculated using eqn (2)–(5) and the results of static contact angle measurements. The dispersive components of surface energies for all functionalized surfaces are about the same. Dispersive components of surface energies arise from random fluctuations in the electron density that lead to temporary dipole interactions. As such, the dispersive component does not depend strongly on molecular structure. Thus, the differences in total surface energies are mainly due to the differences in their polar components, which are attributed to the dipole moments of different organofunctional groups.^31^ Silane coupling agents with small dipole moments, such as BTES and DTES, exhibit a much smaller polar component of their surface energies due to aromatic rings (for BTES) and long alkyl chains (for DTES). For model surfaces containing polar groups, such as APTES-functionalized surfaces, larger polar components of surface energies are observed.

**Fig. 5:**
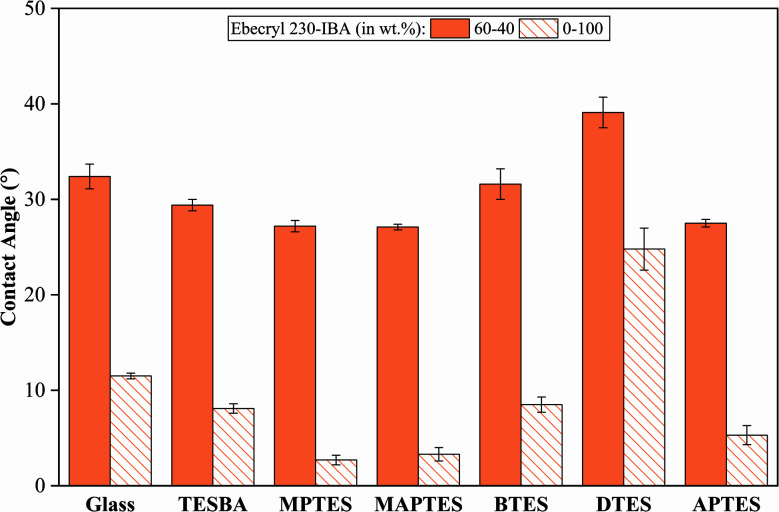
Static contact angles for the formulated resin and pure IBA on glass and functionalized surfaces. Error bars represent standard deviation. *n* = 10 for each condition.

Fig. 5 shows contact angles of the urethane acrylate-based thermosetting resin and pure isobornyl acrylate (IBA) on glass and model functionalized surfaces. In general, while pure IBA is more wetting on all the surfaces than the thermosetting resin, it appears that the urethane acrylate also contributes to the wettability of the resin system. Contact angles for thermosetting resins on all the surfaces are much higher than those for pure IBA on the same surfaces.

Using both the pendant and sessile drop methods, the total surface tension of the urethane acrylate-IBA resin system is 32.46 mN m^−1^, and the polar and dispersive components are 3.26 mN m^−1^ and 29.20 mN m^−1^, respectively. Typical acrylate resins have surface tensions between 29 mN m^−1^ and 38 mN m^−1^, so these results are consistent with reported values.^57^ For pure IBA, the total surface tension is 30.14 mN m^−1^, and the polar and dispersive components are 0.15 mN m^−1^ and 29.99 mN m^−1^, respectively.

**Fig. 6:**
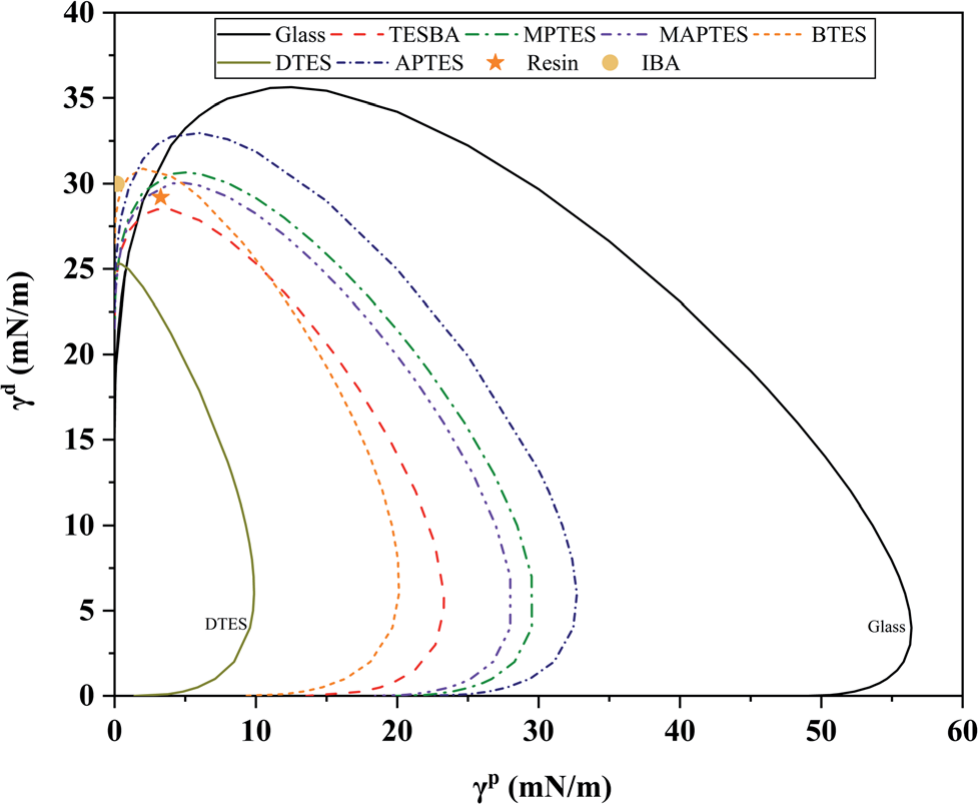
Wettability envelopes of glass and model functionalized surfaces calculated for 0° contact angle.

Wettability envelopes, as shown in Fig. 6, allow for determination of how a liquid will interact with a surface, based on the polar and dispersive components of the liquid surface tension. The thermosetting resin formulation used in this study is located within the wettability envelopes of glass, MPTES-, MAPTES-, BTES-, and APTES-functionalized surfaces, indicating that this resin system can completely wet those surfaces. Nevertheless, while the thermosetting resin shows good wetting towards these surfaces, the wetting is not complete as the measured contact angles are far greater than 0°, as shown in Fig. 5. This discrepancy between contact angles and wettability envelopes can be explained by different migrations of moieties to the contact area between a resin droplet and a substrate since resin is a multicomponent system. IBA is located outside of all the wettability envelopes, indicating that IBA cannot fully wet all the surfaces. This result is consistent with the contact angle analysis shown in Fig. 5.

### Resin characterization

**Fig. 7:**
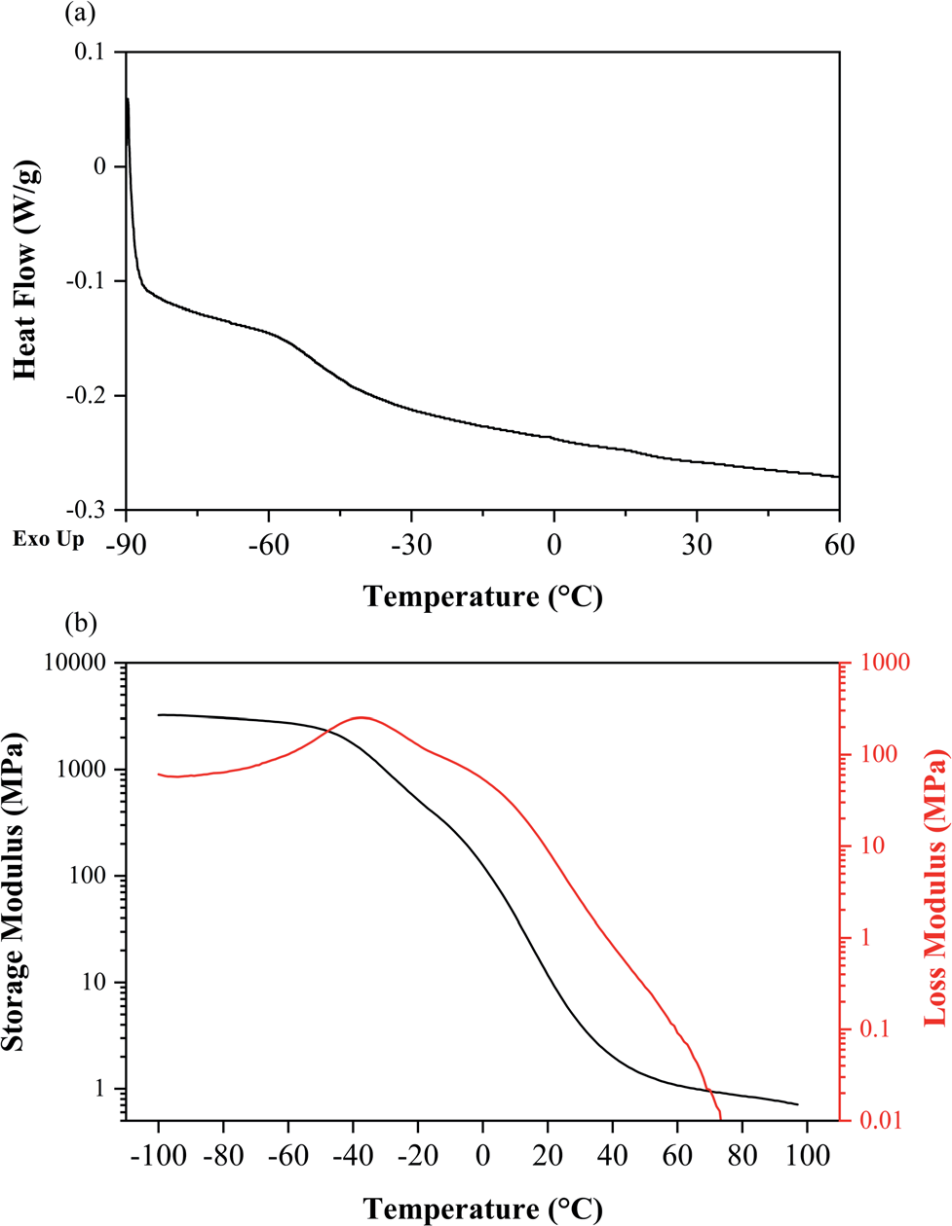
(a) DSC curve of cured thermosetting resin. (b) DMA curves for storage and loss modulus of cured thermosetting resin as a function of temperature.

Fig. 7 shows DSC and DMA curves for the cured urethane acrylate resin. The resin used is a urethane acrylate elastomer with a *T*_g_ of −49.6 °C based on DSC. Such a low *T*_g_ can be beneficial for interfacial interactions between the glass and a polymer because the polymer can remain in its rubbery state even down to very low temperatures. As shown in Fig. 7b, the storage (*E*′) and loss (*E*′′) modulus of the cured resin system at room temperature (20 °C) are 11.74 MPa and 8.94 MPa, respectively. These values are comparable with previous results.^58^

### Interfacial adhesion

**Fig. 8:**
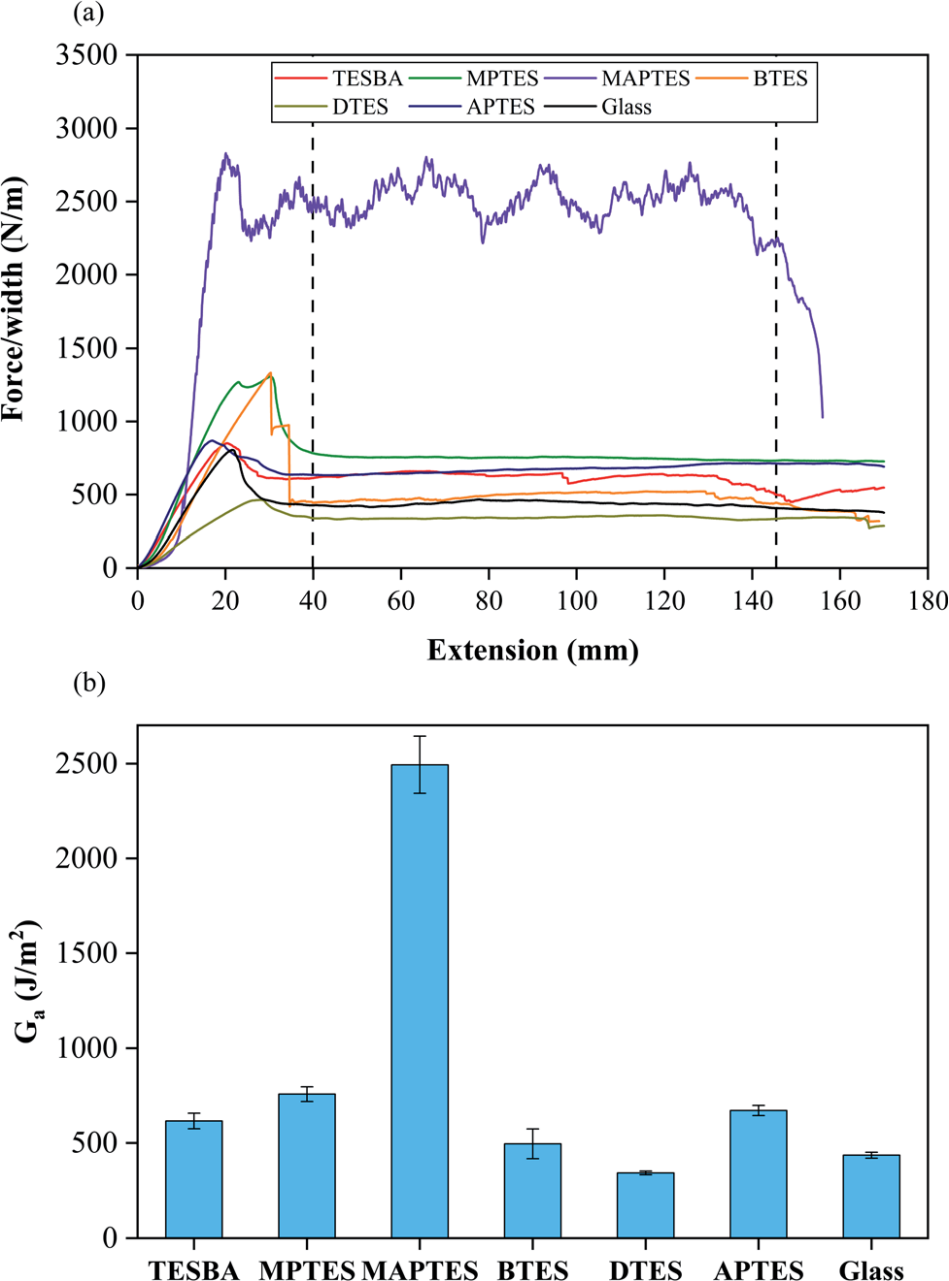
(a) Representative 90° peel test results for glass and model functionalized surfaces. (b) Interfacial fracture energies from the steady-state regions of crack propagation. Error bars represent standard deviation. *n* = 5 for each condition.

90° peel tests were performed to assess the interfacial adhesion between the resin and substrates and the results are shown in Fig. 8.^35^ Interfacial fracture energies are calculated within the steady-state regime, as shown between the two vertical dashed lines in Fig. 8a.

As indicated in Fig. 8b, the interfacial fracture energies vary across different surface chemistries. This variation is due to the existence of connector molecules with different organofunctionalities formed at the glass-thermoset interface, which can either promote or inhibit adhesion.^59^ The highest measured interfacial fracture energy is measured from the MAPTES-functionalized surface. We attribute this high value to covalent bonding between methacrylate groups on the MAPTES-functionalized surface and acrylate groups from the resin.^60,61^ The lowest interfacial fracture energy is observed from the DTES-functionalized surface whose long alkyl chains preclude covalent or non-covalent bonding with the resin. The MPTES- and APTES-functionalized surfaces possess relatively higher interfacial fracture energies. While covalent bond formation is possible between acrylates and MPTES or APTES functional groups, these reactions are unlikely. The thiol groups of MPTES-functionalized surfaces can react *via* thiol–ene photopolymerization with vinyl groups, while secondary amines from APTES-functionalized surfaces can react *via* Michael addition with the same vinyl groups.^60,62^ Formation of covalent bonds between MPTES and acrylates *via* thiol–ene photopolymerization is unlikely because it requires initiation of the thiol group by a free radical. However, free radicals formed through initiation of TPO are far more likely to collide with a vinyl group in the resin phase than a substrate-bound thiol group, especially since TPO is a component within the urethane acrylate resin. Michael addition cannot proceed under these reaction conditions since amines are required to act as nucleophile and base catalyst, but amines are bound to the glass surface for APTES-functionalized surfaces and thus spatially localized to that interfacial region. The interfacial fracture energies for MPTES- and APTES-functionalized surfaces are less than half of that for MAPTES-functionalized surface, which is consistent with few, if any, covalent bonds formed under these reaction conditions.

**Fig. 9:**
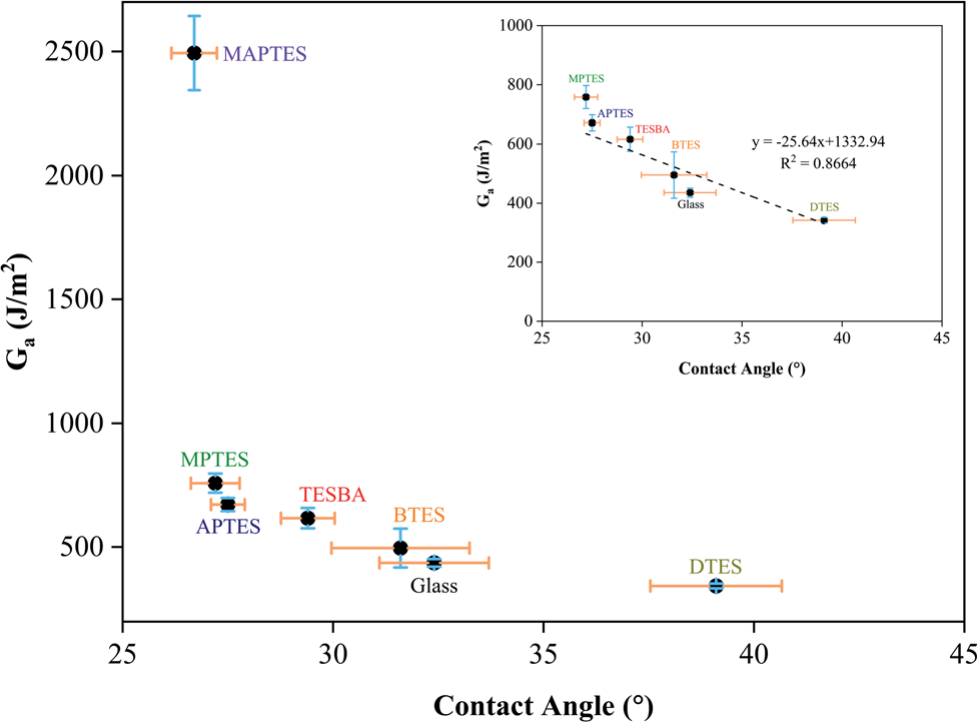
Relationship between interfacial fracture energy and static contact angles of uncured resins on glass and model functionalized surfaces.

The potential to combine surface wettability and interfacial fracture energy provides a new path to design new thermosetting composites with tailorable interfacial adhesion. As shown in Fig. 9, the interfacial fracture energy is positively correlated to the resin wettability and negatively correlated to the static contact angle for of the thermosetting resin. As shown in Fig. 6, wettability envelopes on their own are not sufficient to accurately predict the wetting behavior of the thermosetting resin on glass and model functionalized surfaces. Hence, in this discussion, wettability refers to the wetting behavior instead of the behavior predicted by wettability envelopes. The results shown in Fig. 9 indicate that higher resin wettability on glass and model functionalized surfaces (*i.e.*, lower contact angle) can lead to increased interfacial fracture energy. Interfacial fracture energy is related to the surface energies of both materials at the interface. As the surface tension of the resin is constant in this work, interfacial fracture energy is directly related to the resin wetting behavior, which is controlled by the substrate surface energy. When non-covalent interactions dominate adhesion, a linear correlation between resin contact angle and interfacial fracture energy is observed (*y* = −25.64*x* + 1332.94, *R*^2^ = 0.8664). Based on this linear correlation, increasing the wettability by 5° would result in an increase in interfacial fracture energy of approximately 128.20 J m^−2^. This information can be used to tailor adhesion and to create stimuli-responsive reversible adhesion with greater precision. When covalent bonding occurs at the interface, the interfacial fracture energy is dominated by these bonds.

## Conclusions

We have successfully created a series of model functionalized surfaces using silane coupling agents of different organo-functionalities. While wettability envelopes can be useful tools to predict the interactions between a liquid and a surface, our results demonstrate limitations to this approach. Specifically, multicomponent liquids such as the formulated resin system used here are not well-represented by wettability envelope analysis because different components can preferentially migrate to the surface depending on the substrate surface functionality. The positive correlation between the surface wettability (wetting of resin on substrates), and interfacial fracture energy is observed, which can be used as a powerful tool for screening polymers and solids, whether reinforcements or adherends, for the design novel polymeric composites and adhesively bonded structures.

Overall, this work provides a simple and convenient approach to tailor adhesion. While the current work focuses on adhesion between a soft polymer and stiff substrate, the correlation may be extensible to a wide range of interfaces including those in high-performance reinforced composites.

## Author contributions

Conceptualization: Y. W., E. J. R., A. M. P.; funding acquisition: C. J. H., A. M. P.; methodology: Y. W., C. W., E. J. R., A. M. P.; formal analysis, investigation: Y. W., C. W.; project administration: C. J. H., A. M. P.; supervision: C. J. H., A. M. P.; writing – original draft: Y. W.; writing – review & editing: C. J. H., C. W., A. M. P.

## Conflicts of interest

There are no conflicts of to declare.

## Supplementary Material

RA-011-D1RA05916E-s001
