# Supplementary material for: Effect of surface wettability on the interfacial adhesion of a thermosetting elastomer on glass
Source: RSC Adv. 2021 Sep 21;11(49):31142–51. doi: 10.1039/d1ra05916e (PMC9041377; doi:10.1039/d1ra05916e)
Supplement: RA-011-D1RA05916E-s001 [file RA-011-D1RA05916E-s001.pdf]

Electronic Supplementary Material (ESI) for RSC Advances.
This journal is © The Royal Society of Chemistry 2021

Supporting Information

Effect of surface wettability on the interfacial adhesion of a thermosetting elastomer on glass

Preparation of peel test specimens

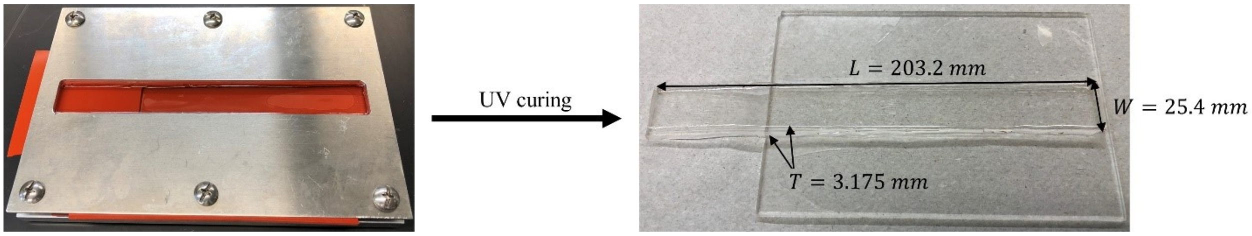

Fig. S1: Curing thermosetting resins onto the substrates to create peel test specimens.

Fabrication of peel specimens for MAPTES-functionalized surfaces

Borosilicate glass plates (McMaster-Carr) were used as the substrates for surface functionalization. Fiberglass fabric with a 2 oz. per square yard weight (Fibre Glast Developments Corporation) was used as the reinforcement layer for the creation of composite specimen. Methacryloxypropyltriethosysilane (MAPTES, Gelest) was used as received for fabrication of model functionalized surfaces and functionalization of fiberglass fabric.

Prior to functionalization, borosilicate glass plates were cleaned by passing through a propane flame, then rinsing with acetone, ethanol, and DI water to remove any impurities from processing, storage, or shipping. For fiberglass fabric, the cleaning procedure is the same except without the flame exposure step. A 20-min cycle of UV-ozone cleaning was further applied in a UV ozone chamber. Surface functionalization was achieved through hydrolytic deposition of silane coupling agents. Solutions consisting of 2 wt.% of a silane coupling agent in a 95:5 by

weight ratio of ethanol to water were prepared. For MAPTES solution, acetic acid was further added to adjust the solution pH to 4.5, based on the procedures provided by the supplier. The solution was hydrolyzed for one hour at room temperature, then the cleaned glass surfaces and fiberglass fabric were immersed in the solutions. Following one additional hour of immersion, the glass substrates and fabrics were removed from the solutions, rinsed with pure ethanol for 5-10 seconds, and placed in an oven at 110 ºC for 30 minutes to achieve surface silanization. The solutions were continuously stirred using a magnetic stir bar during hydrolysis and silanol formation.

Specimens for 90º peel tests were fabricated in accordance with ASTM D6862, by curing urethane acrylate resin with MAPTES-functionalized fiberglass fabrics on MAPTES-functionalized surfaces. The components of the thermosetting resin were mixed and degassed for 3 min each at 2000 rpm using a planetary centrifugal mixer. Mixed resins were partially poured into a CNC-machined aluminum mold with precut silicone rubber sheets. Then, a single layer MAPTES-functionalized fiberglass fabrics was placed onto the previously deposited resin, before pouring the rest of mixed resin to fully coat the fabrics. Specimens were cured and post-cured using the protocol described in the experimental section of the main text.

Peel testing specimens

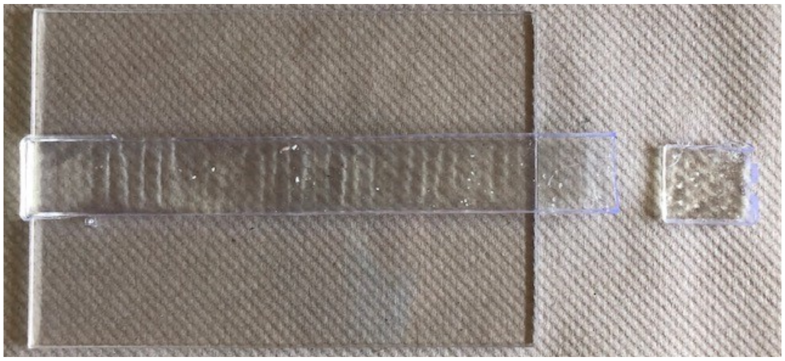

Fig. S2: Failed representative specimen of cured resin on methacryloxypropyltriethoxysilane (MAPTES)-functionalized surfaces during 90° peel test.

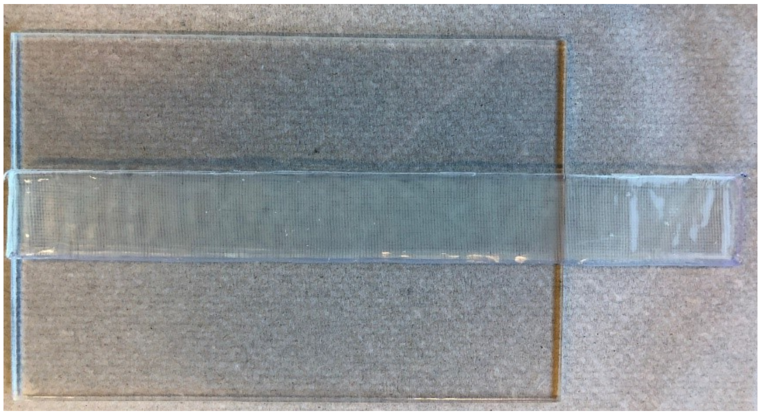

Fig. S3: Representative specimen of cured resin with a single layer of methacryloxypropyltriethoxysilane (MAPTES)-functionalized glass fiber fabrics on MAPTES-functionalized surfaces before 90° peel test.
